# Incidentally discovered Kawasaki disease in an adult man

**DOI:** 10.12669/pjms.37.7.4199

**Published:** 2021

**Authors:** Kamel H. Haider, Sultan Abdulwadoud Alshoabi, Abdulaziz A. Qurashi, Abdullgabbar M. Hamid

**Affiliations:** 1 Kamel H. Haider, Cardiology Department, Prince Mohammed Bin Nasser Hospital, Jazan, Kingdom of Saudi Arabia; 2 Sultan Abdulwadoud Alshoabi, Department of Diagnostic Radiology Technology, College of Applied Medical Sciences, Taibah University, Almadinah Almunawwarah, Kingdom of Saudi Arabia; 3 Abdulaziz A. Qurashi, Department of Diagnostic Radiology Technology, College of Applied Medical Sciences, Taibah University, Almadinah Almunawwarah, Kingdom of Saudi Arabia; 4 Abdullgabbar M. Hamid Radiology Department, Rush University Medical Center, Chicago, IL, United States America

**Keywords:** Adult, Chest pain, Coronary ectasia, Coronary aneurysms, Kawasaki disease

## Abstract

Kawasaki disease (KD) is a systemic vasculitis of unknown cause which usually diagnosed in small children. However, KD can be present as coronary disease in adults even with no history of the disease in childhood. Here, we describe a case of KD in a 42-year-old male patient presented with severe retrosternal chest pain radiating to the left arm and provisionally diagnosed as acute coronary disease. Coronary artery ectasia and multiple aneurysms have been confirmed by coronary angiography that led to the diagnosis of KD. The patient was treated with Aspirin 81 mg orally once daily, Apixapan 5 mg orally twice daily, Rosuvastatin 40 mg orally once daily, Bisoprolol 5 mg orally once daily, and omeprazole 20 mg orally once daily. The patient was improved and discharged with anticoagulant drugs for life. Physicians should be aware that KD can be present as coronary disease in adults even with no history of the disease in childhood and has a limited treatment options due to unfavorable coronary anatomy.

## INTRODUCTION

Kawasaki disease (HD) is an inflammation of the blood vessels throughout the body. The first case was reported by Tomisaku Kawasaki in 1960 who termed it mucocutaneous lymph node syndrome.^1^ It is a self-limiting febrile disease of unknown cause affecting frequently children under five years old.^1^,^2^ According to the fifth revised edition, 2002, of the diagnostic guidelines of KD, the six principal symptoms of KD are: fever persists for five days or more, bilateral conjunctival congestion, changes of lips and oral cavity, polymorphus exanthema, changes of peripheral extremities, and acute non-purulent cervical lymphadenopathy. For diagnosis of KD, five principal symptoms should be available or four principal symptoms in addition to coronary artery aneurysms or dilatation.^2^

In this report, we describe a case of KD in an adult male present with chest pain. This report aimed to elucidate that KD can be present as coronary disease in adults even with no history of the disease in childhood. This report will be importance for physicians to be alert for the presentation of KD in adults.

### Case presentation

A 42-year-old male patient presented to the emergency department with severe retrosternal chest pain radiating to the left arm for two hours. The pain was associated with sweating and vomiting. Patient was a febrile with no cough, no dyspnea and no other cardiac symptoms. The patient is smoker (I pack/day for 20 years), no hypertension, or diabetes mellitus. He has a past history of right nephrectomy due to renal stones.

On examination the patient was conscious, oriented with heart rates of 80 beats/minute, respiratory rate of 17 cycle/minute, and blood pressure= 118/80 mmHg. Normal oxygen saturation (SpO2= 100%), normal jugular venous pressure and no carotid bruits. Chest: normal vesicular breathing. Heart: normal first and second sounds with no additional sounds. Abdomen: soft, no organomegaly, no bruit at renal artery site auscultation. Lower limbs were intact with normal pulse and no edema.

### Lab investigations:

**Fig.1:**
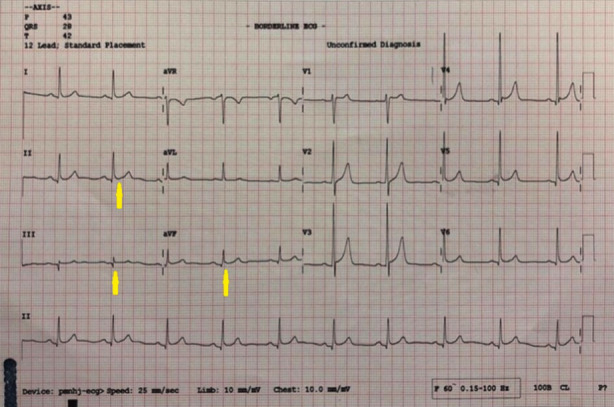
Electrocardiogram (ECG) shows normal sinus rhythm with ST elevation in inferior leads (arrows).

Troponin I was 40.53 ng/ml (Normal range from 0 to 0.04 ng/ml). Serum glucose was 7.12 mmol/L (Normal range from 4.10 to 6.60). Serum creatinine was 101 micromoles/L (Normal range from 39 to 110). Hemoglobin was 13.9 gm/dL (Normal range from 12 to 16). White blood cells were 9.700/mm^3^ (Normal range from 4.500 to 11000). Cholesterol was 5.4 mmol/L (Normal range from 3.6 to 5.2). Electrocardiogram (ECG) showed normal sinus rhythm with ST elevation in inferior lead (Fig. 1). Based on clinical manifestations and ECG findings, the patient was diagnosed as inferior myocardial infarction (MI) and was given thrombolytic drugs as the following: Reteplase 10 units intravenously over two minutes followed by other 10 units over 30 minutes. The patient referred for coronary angiography (CAG).

**Fig.2:**
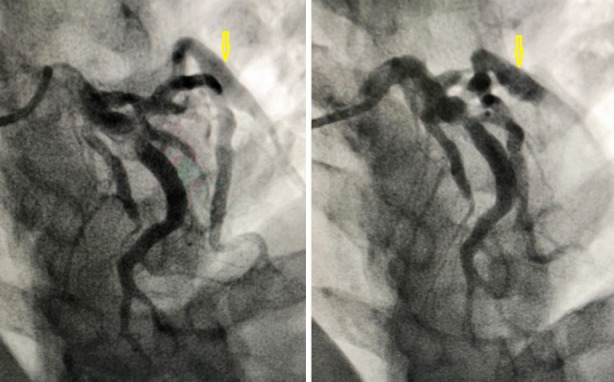
Selected images of left coronary angiogram in anteroposterior cranial view shows diffuse ectasia of the left anterior descending (LAD) artery with aneurysm in the obtuse marginal (OM1) branch, (arrow).

**Fig.3:**
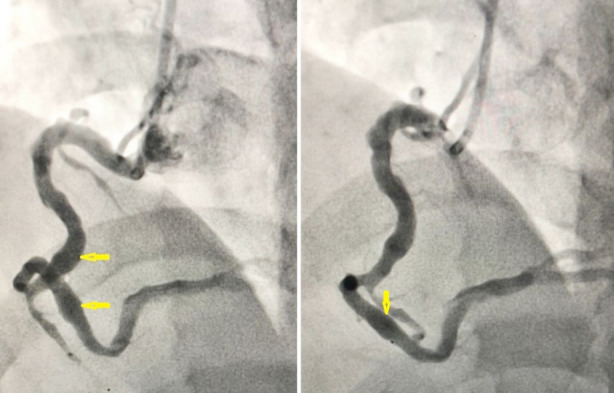
Selected images of right coronary angiogram in cranial views shows right coronary artery (RCA) ectasia with multiple aneurysms in the proximal and middle parts (arrows).

CAG was done within the same day that revealed diffuse ectasia and aneurysmal dilatation of the proximal part of the left anterior descending (LAD) coronary artery, ectasia of the left circumflex artery (LCX), with aneurysm in its obtuse marginal branch (OM1) (Fig. 2). Furthermore, there was diffuse ectasia and multiple small aneurysms in the proximal and mid parts of the right coronary artery (RCA) but no complete occlusion (Fig.3). Further investigation using magnetic resonance angiography (MRA) of the aorta and peripheral arteries shows normal findings. Based on these findings, a diagnosis of Kawasaki disease was made.

According to the guidelines of the european society of cardiology (ESC) for the management of acute MI in patients presenting with ST-elevation^3^, the patient was treated with Aspirin 81 mg orally once daily, Apixapan 5 mg orally twice daily, Rosuvastatin 40 mg orally once daily, Bisoprolol 5 mg orally once daily, and omeprazole 20 mg orally once daily. The patient was improved and discharged with anticoagulant drugs for life.

## DISCUSSION

Kawasaki disease is a systemic vasculitis of unknown cause and usually diagnosed in small children by the presence of five principal symptoms or four principal symptoms in addition to coronary artery aneurysms or dilatation. However, in 10% of cases, it does not fulfil five principal symptoms in those diagnosed with KD, in which KD become a diagnosis of exclusion.^2^ In the current case, coronary artery ectasia and aneurysms have been confirmed by CAG in the proximal part of the LAD coronary artery, and the LCX in an adult man that led to suspect the diagnosis. This case is consistent with Ataya et al. who reported that most of the reported cases of KD in adults was diagnosed based on findings of diffuse coronary ectasia without recall any childhood KD and he also reported that coronary artery ectasia (CAE) usually affects only one or two coronary vessels.^4^

MRA of the thoracic aorta with its branches and abdominal aorta with its branches was normal. No aneurysm in the aortic arch, axillary artery, renal artery or other arteries. Kato et al. reported that 2.2% of KD patients have systemic artery aneurysms and axillary arteries are common sites of aneurysms. However, in the current case, we did not find aneurysms in any peripheral artery.^5^

The current case presented as acute MI and investigations showed diffuse ectasia of the LAD artery, LCX artery and RCA with multiple aneurysms apart from normal distal parts. This is consistent with a previous study by Abugroun et al. who reported that CAE is bad finding in KD that predisposes to worse complications such as thrombus formation, arterial spasm, spontaneous arterial dissection and MI. The presence of coronary artery damage is the hallmark of KD. Abugroun et al. reported that, the most common sites of coronary aneurysms with CAD are the proximal and middle parts of the RCA, the LAD and LCX. Characteristic findings suggesting KD on CAG include proximal ectasia then sudden transition to normal distal segments of the arteries. All of these findings were found typically in our case. Unfortunately, in patient with ectatic infarct-related artery like the current case, percutaneous intervention and stent placement has limited role in treatment due to unfavorable anatomy. Furthermore. There are no obvious guidelines regarding the use of dual antiplatelet therapy or triple therapy and the accurate duration for each.^6^ Further studies about these topics are recommended.

## CONCLUSION

Kawasaki disease is a systemic vasculitis of unknown cause usually diagnosed in small children which can be present as coronary disease in adults even without recall any childhood history of the disease and with no other findings.

### Learning points:

1. Physicians should be aware that KD can be present as coronary disease in adults even with no history of the disease in childhood.
2. KD presents as coronary artery disease in adults with diffuse ectasia and multiple aneurysms has limited treatment options.

### Author’s Contribution:

**KHH** did coronary angiography for the patient and collected data.

**SAA** wrote the initial and approved the final draft of the manuscript and is responsible for accuracy of the work.

**AAQ** revised the manuscript and edited language.

**AMH** revised the final draft of the manuscript.
